# Association of Soy and Exclusive Breastfeeding With Central Precocious Puberty: A Case-Control Study

**DOI:** 10.3389/fendo.2021.667029

**Published:** 2021-07-05

**Authors:** João Soares Felício, Angélica Leite de Alcântara, Luísa Corrêa Janaú, Lorena Vilhena de Moraes, Maria Clara Neres Iunes de Oliveira, Manuela Nascimento de Lemos, Norberto Jorge Kzan de Souza Neto, João Felício Abrahão Neto, Wanderson Maia da Silva, Ícaro José Araújo de Souza, Nivin Mazen Said, Gabriela Nascimento de Lemos, Giovana Miranda Vieira, André Salim Khayat, Ândrea Kely Campos Ribeiro dos Santos, Natércia Neves Marques de Queiroz, Ana Carolina Contente Braga de Sousa, Márcia Costa dos Santos, Franciane Trindade Cunha de Melo, Pedro Paulo Freire Piani, Karem Miléo Felício

**Affiliations:** Endocrinology Division, University Hospital João de Barros Barreto, Federal University of Pará, Belém, Brazil

**Keywords:** precocious puberty, soy, endocrine interferer, exclusive breastfeeding, infantile nutrition

## Abstract

**Introduction:**

While soy is suggested as a possible risk factor, exclusive breastfeeding (EBF) has a likely protective effect in precocious puberty. Our aim was to evaluate the association between both of these variables with central precocious puberty (CPP)

**Methods:**

We performed a retrospective, case-control study. A total of 161 girls were divided into two groups: 84 patients diagnosed with CPP composed the case group and 77 patients without the diagnosis of CPP (had gone through normal onset of puberty) were the control group.

**Results:**

Our control group had a higher presence of EBF >6 months, which was an important protective factor for CPP (OR: 0.5; IC 95%: 0.3–0.9, p = 0.05) and also correlated negatively with the presence of it (r = −0.2; p < 0.05). Oppositely, the use of soy was significantly higher in the CPP group, (OR: 3.8; IC 95%: 1.5–6, p < 0.05) and positively correlating (r = 0.2; p < 0.01) with the presence of CPP. Duration of soy intake (years) correlated with bone age (r = 0.415; p < 0.05). A logistic regression was performed to evaluate the effects of EBF duration and soy on CPP. The model was significant (x² (2) = 20,715, p = <0.001) and explained 12.2% (Nagelkerke R2) of the variance, correctly classifying 62.5% of cases. EBF was associated with a reduction of likelihood of having CPP [OR = 0,187 (CI = 0.055–0,635); Wald = 7,222, p = 0.007], while soy intake increased the risk [OR = 3.505 (CI) = 1,688–7,279, Wald = 11,319, p = 0.001].

**Conclusion:**

Our data found the use of soy was associated with CPP. Additionally, EBF was pointed as a protective factor. However, future prospective studies are needed to clarify this issue.

## Introduction

Puberty is a complex biological process, and its timing has substantial physical, psychosocial, and long-term health implications for the pediatric population (1, 2). Central precocious puberty (CPP) is often defined as pubertal onset before 8 years of age in girls and before 9 years of age in boys (although ages can vary among populations to define this condition), and it can be responsible for early progression of secondary sexual characteristics, rapid bone maturation, reduced final height, inappropriate body appearance, and psychological behavioral abnormalities (3, 4).

Its course can be determined by a series of endogenous and exogenous factors, such as environmental endocrine interferers. Soy is suggested as a risk factor because it contains isoflavones, a group of phytoestrogens composed of three main substances (genistein, daidzein, and glycitein) with chemical structure and hormonal activity similar to estradiol (5, 6). However, it is still a controversial topic, as several authors have attested the safety of their usage in infant feeding (7–9).

On the other hand, exclusive breastfeeding (EBF) has shown to be a likely protective factor for CPP (10, 11). According to the World Health Organization (12), infants should be exclusively breastfed in the first 6 months of life and partial breastfeed until 2 years of age. Human milk can provide short and long-term health benefits, such as promotion of brain development, protection against infection diseases, type II diabetes, and overweight (13). Thereby, breastfeeding as a protective factor for obesity could also indirectly protect from the development of CPP.

Thus, due to the remaining controversy, this study aimed to evaluate the association between EBF and soy feeding with CPP.

## Materials and Methods

### Study Design and Patients

We performed an observational, retrospective, case-control study to evaluate the relationship between the occurrence of CPP, EBF, and consumption of soy during early childhood. This study was developed according to the Declaration of Helsinki and Nuremberg Code and was approved by the University Hospital João de Barros Barreto ethics committee.

A total of 161 subjects were divided into two groups: 84 female patients diagnosed with CPP composed the case group. Others 77 girls without the diagnosis of PP (had gone through normal onset of puberty) were the control group. They came to evaluate other pathologies or investigate precocious puberty, but it was classified as normal and they were followed through all puberty. In our Pediatric Endocrinology division, the evaluation of bone age is a standard procedure while investigating children with symptoms of short stature or any alteration in pubertal development, so we were able to acquire this information from control group. Their bone age was measured only in the first visit, for diagnosis purposes and alongside other exams needed to exclude precocious puberty. Once we confirmed that their growth was normal, bone age was not performed routinely, so, comparison purposes, we used the one obtained in the initial visit. There were no Afro-American nor Latin groups. Patients that during the follow-up used systemic corticosteroids more than four times a year, with uncontrolled hypothyroidism, diabetes mellitus, growth hormone deficiency, peripheral precocious puberty, chronic and/or inflammatory diseases, genetic syndromes, malnutrition, and family history of PP were excluded from both groups.

The diagnosis of CPP and decision to treat with Gonadotropin Release Hormone (GnRH) analog was based on the current guidelines (14–17), which uses as clinical criteria in girls: breast development, with or without pubarche/axilarche before 8 years old. All children diagnosed with the condition were treated using GnRH analogs. All CPP group had normal skull magnetic resonance image (MRI) and performed GnRH stimulation test. In addition, all tests needed to exclude other causes of PP were performed. Finally, all patients underwent pelvic ultrasonography.

### Clinical and Laboratorial Data

Data were collected from medical records of patients attended in endocrinology and pediatric services of the HUJBB from January 2010 to December 2018. The following information were collected from each patient: age (years); ethnicity; bone age; height (cm); weight (kg); serum levels of follicle stimulating hormone (FSH) and luteinizing hormone (LH), measured in (IU)/L (18); pubertal stage classified by Tanner method (19); age of mother at menarche; presence, duration, and type (exclusive, complementary, or mixed) of breastfeeding; frequency, period of use, and type of soy infant formula; age of onset of secondary sexual characters (thelarche, pubarche, and axilarche), and age of menarche.

The time that basal data were collected differed between case and control group. For comparison, we intended to evaluate all patients during puberty. In case group, data were addressed during clinical visits in which CPP was diagnosed. In control group, it occurred at the first clinical visit after onset of puberty. Therefore, as expected, case group was younger than control group, but both had initiated their pubertal development.

Patients’ weight and height were measured in triplicate using the Harpender Stadiometer during clinic examination. The bone age was based on the analysis of left hand and wrist radiographs, using Greulich & Pyle’s standard method (20). Body mass index (BMI) was calculated as weight/(height²). Tanner method was used for pubertal staging (19). Children who consumed soy did so because of allergies. To quantify the amount of soy consumed by the child, we checked medical records from each patient. In our pediatric endocrinology department, we routinely question about soy intake and record it. An estimate was made based on the protein content of the soy-based formulas or soy food (21–23), frequency and volume of daily intakes (100 ml for baby bottle or 200 ml for glass, which was confirmed by the caregiver).

The data collection was carried out between March 2016 to May 2019.

### Statistical Analysis

Data concerning clinical and epidemiological characteristics were processed using descriptive statistics. Continuous variables with normal distribution are presented as Mean ± Standard Deviation, and those with non-normal distribution are shown as median and interquartile range (IQR). To establish the relationship between risk factors, linear and logistic regression models were created. The logistic regression aimed to clarify if the presence of EBF >6 months (yes/no) and use of soy (yes/no) had an independent influence on CPP. We defined the occurrence of CPP as the dependent variable and included both cited above as independent variables in our model, which was adjusted for BMI Z score. All tests were performed using the SPSS Statistics 22^®^ software (IBM Corp., Armonk, NY, USA). Results were considered significant if p-value <0.05.

## Results

**Figure 1:**
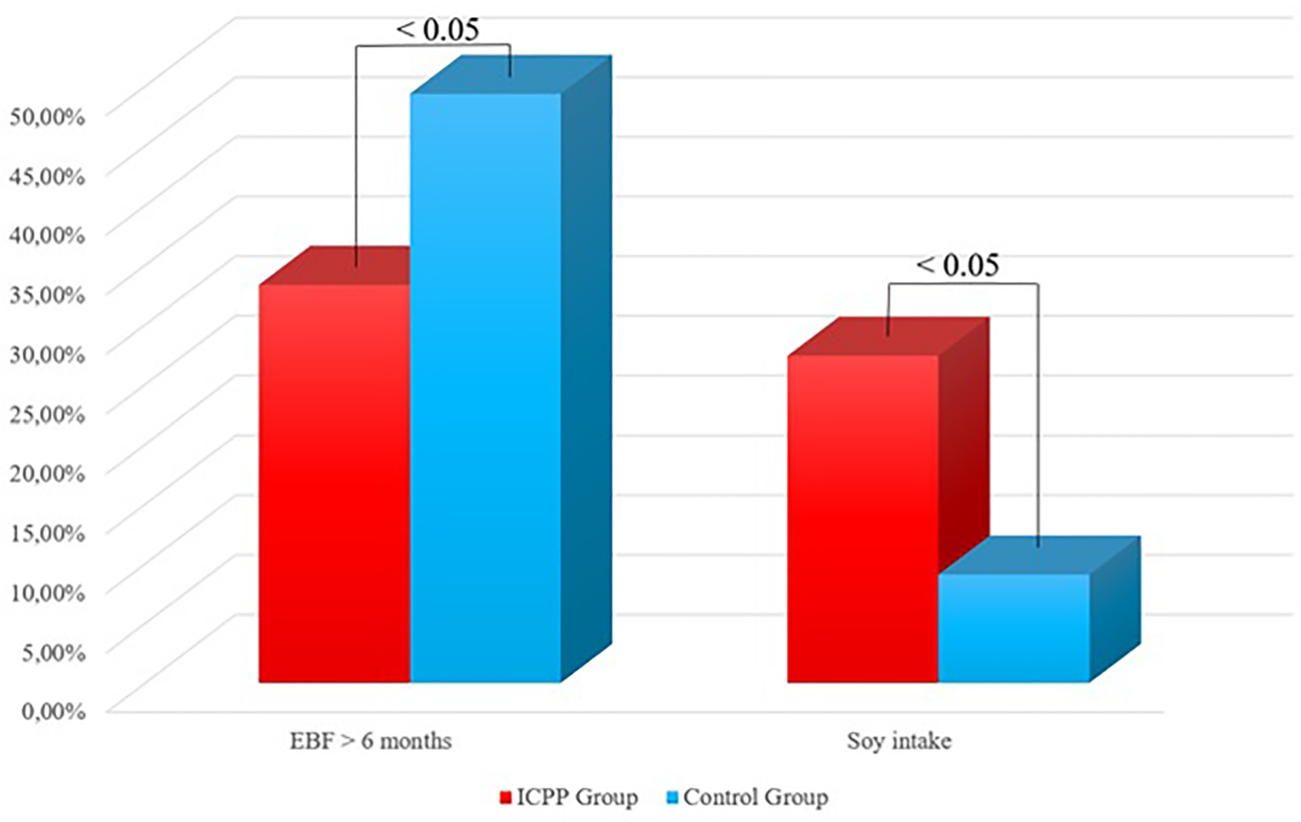
Comparison regarding EBF and soy intake between groups.

At the time of CPP diagnosis, patients in PP group had an increase of 2.0 ± 1.6 years in bone age, and LH and FSH levels were 0.3 ± 0.7 (mIU/ml) and 2.4 ± 1.4 (mIU/ml), respectively. In control group, there is no advance in bone age. We compared pubertal characteristics and (thelarche, pubarche, and menarche), risk factors for CPP (mother’s menarche), and diet history (EBF and soy-based formulas) between both groups, showed in **Table 1** and **Figure 1**.

**Table 1:** Clinical characteristics and risk factors.

Characteristics	Total (N = 161)		
	PP (N = 84)	Control(N = 77)	p-value
**Age (years)**	7.9 ± 1.8	10.0 ± 2.1	<0.001
**BMI-SDS**	1 ± 1.4	0.5 ± 1.2	<0.05
**Thelarche (years)**	7.2 ± 1.8	9.4 ± 2.2	<0.001
**Pubarche (years)**	7.3 ± 1.7	9.1 ± 2.7	<0.001
**Menarche (years)**	12.8 ± 1.3	10.8 ± 0.7	<0.005
**Mother’s menarche (years)**	12.2 ± 1.6	11.7 ± 1.1	NS (0.128)
**EBF duration (years)**	0.3 ± 0.2	0.4 ± 0.3	<0.05
**EBF >6 months (yes, %)**	28 (33%)	38 (49%)	0.05 (OR: 0.5; IC 95%:0.3–0.9)
**Use of soy-based formulas (yes, %)**	23 (28%)	7 (9%)	<0.05 (OR: 3.8; IC 95%: 1.5–9)

PP, precocious puberty; BMI-SDS, body mass index standard deviation score; EBF, exclusive breastfeeding; NS, not significant.

Patients’ soy consumption is shown in **Table 2**.

**Table 2:** Characteristics of soy intake in females.

	Girls exposed to soy/total (N = 30/161)		
	PP (N = 23/84)	Control (N = 7/77)	p-value
**Daily amount of soy intake (g/day)**	8.9 ± 5.4	7.2 ± 5.4	<0.05
**Total amount of soy intake (kg)**	11 ± 11	4.9 ± 5.6	<0.05
**Duration of soy intake (years)**	3.0 ± 2.3	1.8 ± 1.6	NS (0.182)
**Age of first soy intake (years)**	1.3 ± 1.6	2.7 ± 3.3	NS (0.588)
**Frequency of daily soy intakes**	2.3 ± 1.0	1.6 ± 1.3	NS (0.133)

NS, not significant.

Correlations between possible risk factors and presence of CPP are shown in **Table 3**.

**Table 3:** Correlations between presence of CPP and risk factors in girls.

Risk Factor	r	p
**Use of soy-based formula (yes/no)**	0.2	<0.01
**Duration of EBF (years)**	−0.2	<0.05
**BMI-SDS**	0.2	<0,01
**EBF >6 months (yes/no)**	−0.2	<0.05
**Total amount of soy intake (kg)**	0.3	<0.0001
**Daily amount of soy intake (g/day)**	0.3	<0.0001
**Mother’s menarche (years)**	0.2	0.06

EBF, Exclusive breastfeeding; BMI-SDS, Body mass index standard deviation score.

**Figure 2:**
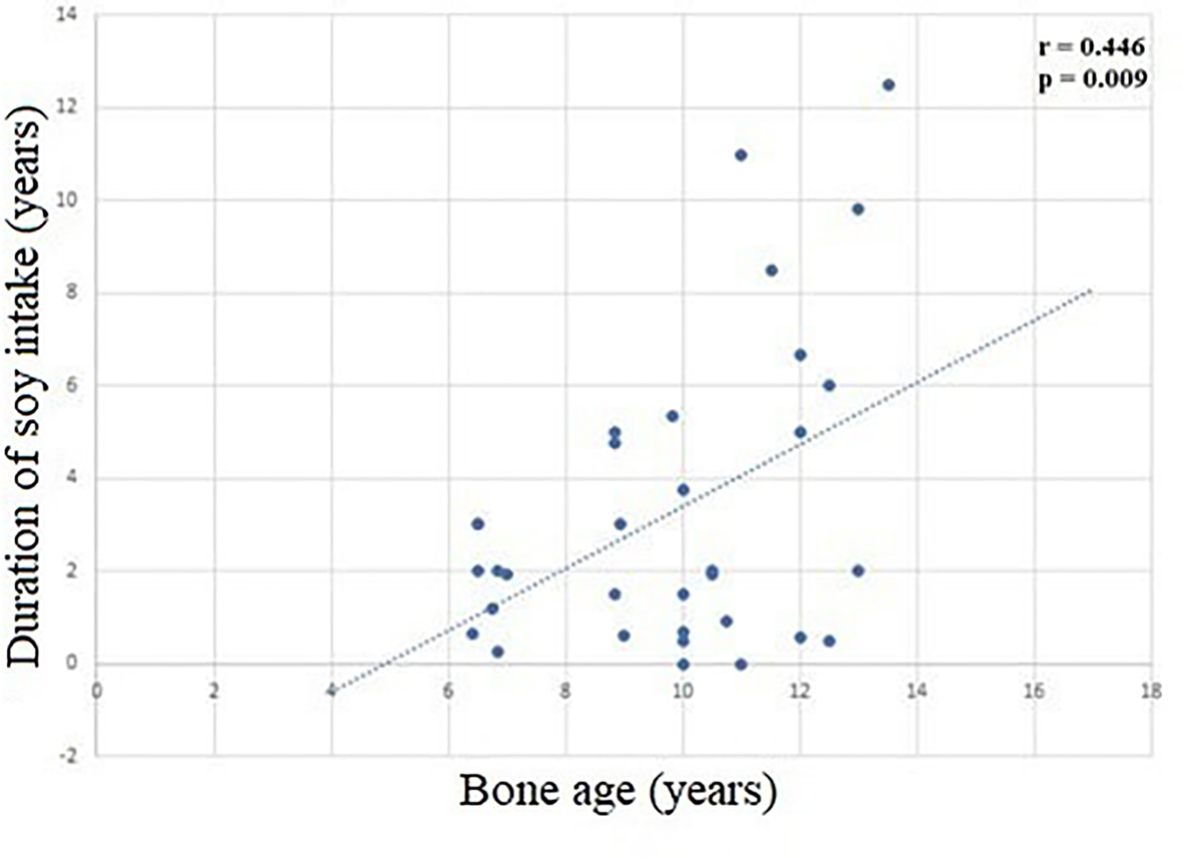
Correlation between duration of soy intake e and bone age (r = 0.446, p = 0.009).

When all groups were evaluated, we also found a correlation between duration of soy intake and bone age (r = 0.415, p < 0.05), as shown in the **Figure 2**.

Finally, a logistic regression was performed to address the effects of presence of EBF for at least 6 months (yes/no) and use of soy (yes/no) on the likelihood that participants develop PP. We defined the occurrence of PP as the dependent variable and included both as independent variables in our model. The logistic regression model was statistically significant (x² (2) = 14.45, p = 0.001). The model explained 11.5% (Nagelkerke R2) of the variance and correctly classified 62.5% of cases. EBF >6 months was associated with a reduction of likelihood of having PP (OR = 0.476 (CI = 0.245–0.924); Wald = 4.813, p = 0.03), while soy intake increased the likelihood of having this condition [OR = 3.974 (CI) = 1.571–10.054, Wald = 8.487, p = 0.004]. Our regression was adjusted for BMI Z score, which was not selected for the model.

## Discussion

We have found that the use of soy was associated with CPP. Additionally, EBF was associated with a degree of protection in our subjects.

Some authors attested soy’s safety (7–9, 24, 25). Andres et al. (24) led a cohort study with 101 pediatric patients, which were grouped according to its infant diet (breastfed and soy formula). They assessed their reproductive organ size by ultrasonography at age of 5 years old and found no difference between groups. However, their analysis did not last until patients’ puberty. Another prospective study (26) followed 1,239 girls aged 6–8 years in the USA, and found no association between puberty and urinary excretion of isoflavones. Nevertheless, that study did not evaluate specifically patients with CPP. In 2011, a panel of 14 independent scientists stated that there was a minimal concern about safety of soy-based infant formula, but they recognized the need for long-term data to verify this issue (27).

Korean girls tend to begin menstruating at younger ages, and two case-control studies (28, 29) found that urinary isoflavone and genistein are increased in patients with PP compared to controls. Nevertheless, they have showed some weaknesses. Both of them did not perform MRI to rule out central nervous system (CNS) lesions as causes of CPP, and it is well-known that up to 20% of all central PP cases in young girls are not idiopathic (28–31). In addition, they chose control group among children who visited the clinic at same period with no pubertal signs. In our study, the control and case groups were followed during all puberty course and CNS lesions were ruled out with MRI, aside laboratorial tests, in all patients. In fact, our retrospective case-control design is more appropriate to detect this interaction.

Messina et al. (7), in a recent review in Asian populations, suggested that a reasonable intake recommendation for children between 2–12 years old is 5–10 g/day of soy protein. However, most studies analyzed in this review failed to report an accurate soy intake and central precocious puberty’s prevalence. Our data suggest that the daily amount of 8.9 g/day—which is included in Messina’s safety range—is associated to PP.

Sinai et al. (8) in 2019, in a nested case-control study, selected infants from a cohort who were prospectively followed from birth until age of 3 years for eating habits and development of cow-milk allergy, and then were reevaluated. They found no association between puberty and infantile nutrition, after controlling BMI and family data, but the number of patients were too small (29 patients in case group and 60 in control group) and most of case group were boys, in which PP is uncommon (1). This fact possibly influenced their results. As far as we are aware, this is the first case-control study which followed children with CPP and during all course of puberty, analyzing in detail soy consumption.

There are some authors claiming that soy can play a role in early pubertal development, supporting our findings (32–34). In a review, Chakraborty et al. (32) suggested that phytoestrogens play a role in PP. In agreement, Segovia et al. (33) in a cross-sectional study with 248 boys have found that pubarche manifests earlier in those who consume larger amounts of soy. Consonantly, another study (35) examined 166 children and concluded that girls fed with soy-based formula had higher LH levels when compared to the ones that consumed cow-milk. Finally, Adgent et al. (34) prospectively followed 2,920 girls from their intrauterine lives until puberty, and found that the earlier girls are exposed to soy, the earlier they menstruate. Nevertheless, they have not established an increased risk of precocious puberty in this cohort. This issue still needs to be clarified.

Our study also found breastfeeding as a possible protective factor for the onset of precocious puberty, agreeing with many others (10, 11, 36). This may occur due to breastfeeding also being a protective factor for obesity, and obesity itself could interfere with the onset and development of precocious puberty (37, 38), which was also reaffirmed by our study. Li et al. (39), in a 2017 systematic review, and Lian et al. (40), in a research with 2,996 girls aged 9 to 19 years, found that girls with overweight or obesity reached puberty earlier when compared with normal weight girls. In our study, our case group showed an increased BMI, but, according to our regression model, the soy intake was an independent variable associated with the presence of CPP.

Moreover, Kale et al. (11) found that mixed-fed or predominantly breastfed girls showed later onset of breast development compared to formula fed girls, also, the duration of breastfeeding was directly associated with age at onset of breast development. Aghaee et al. (36) in a study with 3,331 girls, found that those not breastfed were more likely to experience earlier thelarche and pubarche compared with girls who were breastfed ≥6 months. Similarly, a prospective observational study with boys and girls found an independent preventive association of breastfeeding for ≥6 months and early pubertal development, reinforcing our findings (10).

A limitation of our study was its design. A large prospective cohort study could be the gold standard to clarify the causal effects of soy intake in precocious puberty. Data regarding serum and urinary isoflavones, socio-economic status, and low birth weight prevalence were not available in our analysis. As we used retrospective data, another limitation is the possibility of inaccuracy, which was minimized by strictly checked medical records from each patient and confirmation of those data with an interview in a posterior moment.

## Conclusion

We have found that use of soy has an association with CPP. An earlier start of consumption and intake duration can possibly play a role in development of disease. Additionally, EBF was pointed as a possible protective factor in our subjects. However, future prospective studies are needed to clarify this issue.

## Data Availability Statement

The original contributions presented in the study are included in the article. Further inquiries can be directed to the corresponding author.

## Ethics Statement

The studies involving human participants were reviewed and approved by University Hospital João de Barros Barreto ethics committee. Written informed consent to participate in this study was provided by the participants’ legal guardian/next of kin.

## Author Contributions

All persons who meet authorship criteria are listed as authors, and all authors certify that they have participated sufficiently in the work to take public responsibility for the content, including participation in the concept, design, analysis, writing, or revision of the manuscript. JF, AA, KF, and LJ took part in conception and design of study. FM, NQ, AS, MS, LM, and MO were responsible for acquisition of data, while NS, IS, WS, NN, and JN have done the analysis and interpretation of data. ML, GL, GV, ÂS, AK, and PP have drafted the manuscript together. All authors contributed to the article and approved the submitted version.

## Conflict of Interest

The authors declare that the research was conducted in the absence of any commercial or financial relationships that could be construed as a potential conflict of interest.

## Abbreviations

BMI, Body Mass Index; CI, Confidence Interval; EBF, Exclusive Breastfeeding; FSH, Follicle Stimulating Hormone; GnRH, Gonadotropin Release Hormone; HUJBB, University Hospital João de Barros Barreto; CPP, Central Precocious Puberty; IQR, Interquartile Range; LH, Luteinizing Hormone; MRI, Magnetic Resonance Image; NS, Not Significant; OR, Odds Ratio; PP, Precocious Puberty; SD, Standard Deviation; WHO, World Health Organization.
